# BEST: a web server for brain expression Spatio-temporal pattern analysis

**DOI:** 10.1186/s12859-019-3222-6

**Published:** 2019-12-05

**Authors:** Liyuan Guo, Wei Lin, Yidan Zhang, Wenhan Li, Jing Wang

**Affiliations:** 1 CAS Key Laboratory of Mental Health, Institute of Psychology, Chinese Academy of Sciences, 16 Lincui Road, Chaoyang District, Beijing, 100101 China; 2 Department of Psycholog, University of the Chinese Academy of Sciences, Beijing, 100049 China; 3 Oumeng V medical Laboratory, Hangzhou, 310013 Zhejiang China

**Keywords:** Web server, Brain expression pattern analysis, Spatio-temporal, Co-expression cluster, Visualization

## Abstract

**Background:**

Dysregulated gene expression patterns have been reported in several mental disorders. Limited by the difficulty of obtaining samples, psychiatric molecular mechanism research still relies heavily on clues from genetics studies. By using reference data from brain expression studies, multiple types of comprehensive gene expression pattern analysis have been performed on psychiatric genetic results. These systems-level spatial-temporal expression pattern analyses provided evidence on specific brain regions, developmental stages and molecular pathways that are possibly involved in psychiatric pathophysiology. At present, there is no online tool for such systematic analysis, which hinders the applications of analysis by non-informatics researchers such as experimental biologists and clinical molecular biologists.

**Results:**

We developed the BEST web server to support Brain Expression Spatio-Temporal pattern analysis. There are three highlighted features of BEST: 1) visualization: it generates user-friendly visual results that are easy to interpret, including heatmaps, Venn diagrams, gene co-expression networks and cluster-based Manhattan gene plots; these results illustrate the complex spatio-temporal expression patterns, including expression quantification and correlation between genes; 2) integration: it provides comprehensive human brain spatio-temporal expression patterns by integrating data from currently available databases; 3) multi-dimensionality: it analyses input genes as both a whole set and several subsets (clusters) which are enriched according to co-expression patterns, and it also presents the correlation between genetic and expression data.

**Conclusions:**

To the best of our knowledge, BEST is the first data tool to support comprehensive human brain spatial-temporal expression pattern analysis. It helps to bridge disease-related genetic studies and mechanism studies, provides clues for key gene and molecular system identification, and supports the analysis of disease sensitive brain region and age stages. BEST is freely available at http://best.psych.ac.cn.

## Background

The well-regulated gene expression in the human brain, the highly heterogeneous and lifelong changing organ, is the molecular basis of normal cognitive and behavioural functions. The dysregulated gene expression patterns in brain have been reported in several mental disorders, such as schizophrenia [1], bipolar disorder [2] and Alzheimer’s disease [3]. Limited by the difficulty of obtaining samples, although excellent epigenetic studies such as the series of works from the psychENCODE project have been reported [4, 5], psychiatry molecular mechanism research still relies heavily on clues from genetics studies.

Currently, several data tools provide abundant RNA data from human brains to support gene expression analysis. The Genotype-Tissue Expression (GTEx) (https://gtexportal.org) provides comparative results of gene expression quantifications in different tissues include human brain [6]; the Expression Atlas (https://www.ebi.ac.uk/gxa/home) provides gene expression quantifications across species and biological conditions [7]. Specific to the human brain, the Allen Brain Atlas (http://human.brain-map.org/) and BRAINSPAN (http://www.brainspan.org/) support heatmap generation to view the gene expression quantifications in multiple brain regions from different donors [8–10]. The Human Brain Transcriptome (HBT, http://hbatlas.org/) provides genome-wide, exon-level transcriptome data generated from both hemispheres of postmortem human brains, and its “Developmental Trajectories” module provides principal component analysis of the expression of grouped genes in six brain regions [11, 12].

By using reference data from the above resources, multiple types of comprehensive gene expression pattern analysis have been performed on psychiatric genetic results [13–16]. In such studies, the expression statuses of interest genes were reviewed in different brain regions (spatial pattern) and age stages (temporal pattern); the potential functional combinations of genes of interest were presented by their enrichment status in co-expression gene clusters, which were generated by Weighted Correlation Network Analysis (WGCNA) [17]. The analysis results can be shown as intuitive graphics, such as expression heatmaps or enrichment heatmaps in a spatial-temporal matrix, a co-expression network constructed by interest genes. These systems-level spatial-temporal expression pattern analyses integrated genetic data with gene expression data of normal adult brains and identified specific brain regions, developmental stages and molecular pathways that are possibly involved in psychiatric pathophysiology. They also provided valuable evidence for further biological and clinical molecular research.

At present, there is no online tool for such systematic analysis, which hinders the applications of analysis among non-informatics researchers, such as experimental biologists and clinical molecular biologists. Thus, we developed the BEST web server to support Brain Expression Spatio-Temporal pattern analysis. This web tool performs gene expression pattern analysis with reference to pre-integrated spatial-temporal expression data generated from healthy human brains. The analysis results are presented as heatmaps, Manhattan plots, networks, and other types of graphics. A user-friendly user interface module was designed for input data submission, browsing results and download.

## Implementation

### Reference data

#### Human single nucleotide polymorphisms (SNPs), genes and brain spatio-temporal expression datasets

**Fig. 1:**
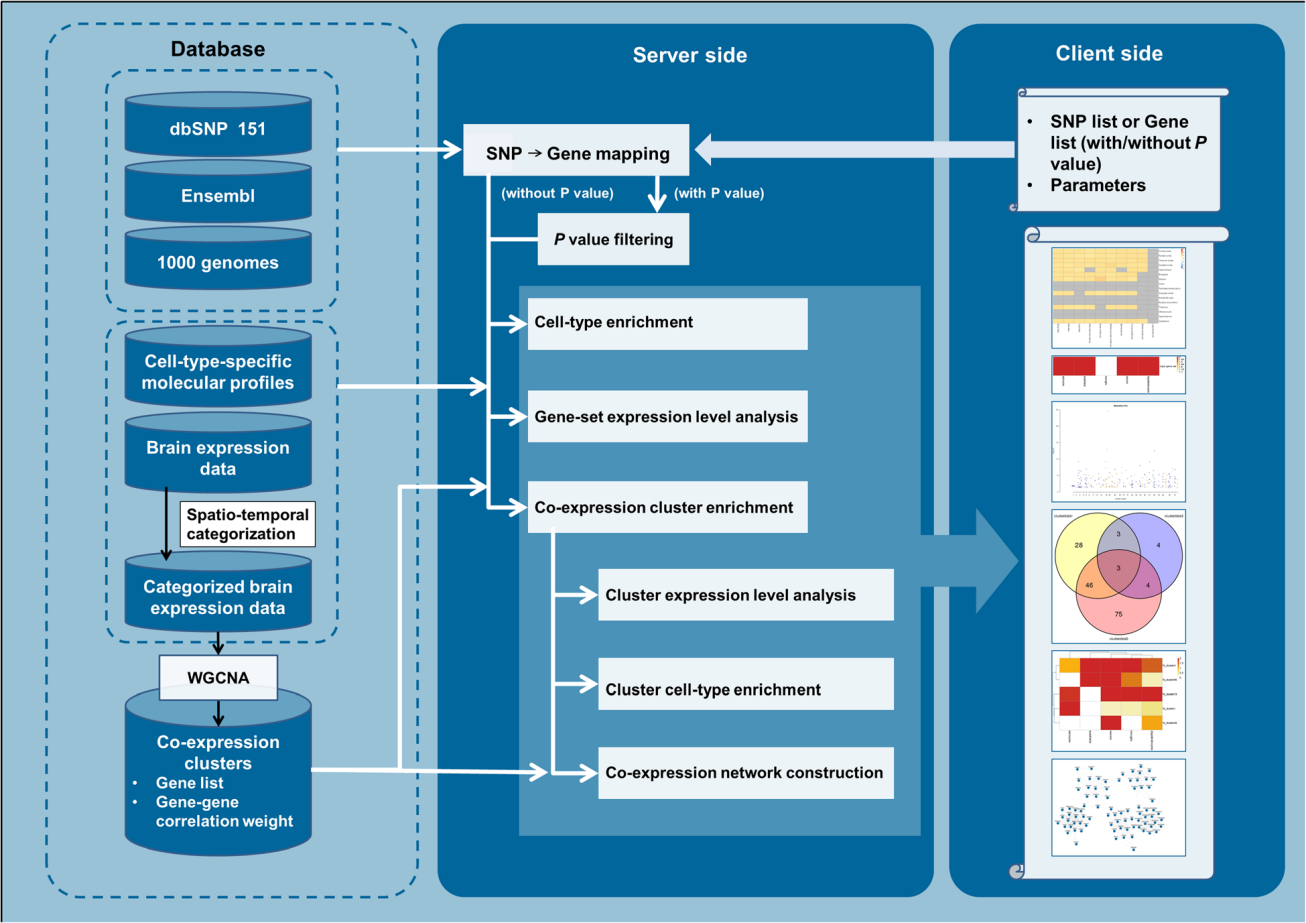
The system architecture and overview of BEST web server

rsID and genomic location information of genome-wide human single nucleotide polymorphisms (SNPs) were obtained from the NCBI dbSNP database (https://www.ncbi.nlm.nih.gov/SNP/, build 151) [18] on the GRCh38 coordinate. Hugo gene nomenclature committee(HGNC) official symbols and genomic locations of genome-wide human genes (both coding genes and non-coding genes) were downloaded from the Ensembl database (www.ensembl.org), assembly GRCh38.p12 [19]. Eight human brain expression datasets were obtained from BrainSpan Atlas [10, 12, 20, 21], Allen brainmap [8], GTEx [6, 22], and other sources [23–26]. As shown in Fig. 1, samples in expression datasets were categorized based on the corresponding brain region and the age of individual from which it was obtained. In each dataset, the average expression quantification of each gene was calculated based on data from all samples in the same spatio-temporal category. The detailed information of reference expression data and the spatio-temporal categories are described in Additional files 1, 2, 3 and 4. In addition to expression data for brain regions, cell-type-specific expression profiles, which provide specific expression gene sets for astrocytes, endothelial cells, microglia, neurons, and oligodendrocytes, were also used in BEST [27].

#### Co-expression modules

Weighted gene correlation network analysis (WGCNA) was performed for five of the eight expression datasets to identify clusters of co-expressed genes [17]. According to the guidelines of the WGCNA R package (Version 1.64–1, http://horvath.genetics.ucla.edu/html/CoexpressionNetwork/Rpackages/WGCNA/), three expression datasets with fewer than eight spatio-temporal categories have not been used in WGCNA analysis. The “blockwiseModules” function in WGCNA was used to construct co-expression networks and detect clusters, and the gene cluster size was maintained between 30 and 5000. The detected co-expression clusters (gene sets and the correlation weights between genes) were stored for subsequent analysis. The statistics of co-expression clusters are shown in Additional files 1, 2, 3 and 4.

### Analysis and statistics

#### SNP mapping and gene-based *P* value calculation

BEST accepts the human gene list and SNP list (with/without *P* value) as input. As shown in Fig. 1, inputs of SNPs are mapped to genes according to their chromosomal locations, and the gene-based *P* value is computed with an F-test based on a multiple linear principal components regression module by using the MAGMA software [28]; the test is corrected by using a user selected method. Four correction methods are provided, including Bonferroni, Sidak, false discovery rate (FDR)-Benjamini Hochberg (BH), and FDR-Benjamini Yekutili (BY). Linkage disequilibrium (LD) between SNPs is fully accounted for during the computation (28), and LD information for five populations, including African (AFR), mixed American (AMR), East Asian (ESA), European (EUR) and South Asian (SAS), was compiled from Phase 3 of the 1000 genomes project [29].

#### Gene set-based brain spatio-temporal expression analysis

Input or mapped genes with *P* values are filtered before expression analysis: genes mapped from inputs of the SNP list with *P* value are filtered by user selected cutoffs for adjusted *P* values; inputs of the gene list with *P* value are filtered by the cutoff of 0.05. Filtered genes are treated as a whole set, and the average expression quantifications are calculated with reference to user selected expression datasets. Inputs or mapped genes without *P* values are analysed without any filter. Expression heatmaps are generated according to the analysis result in a matrix of brain regions by age periods (the spatio-temporal matrix). Except for brain region and age period related spatio-temporal analysis, cell-type-specific gene set enrichment is also performed by using Fisher’s exact test with the cutoff of 0.05, and an enrichment heatmap is generated accordingly.

#### Co-expression gene cluster enrichment and co-expression network construction

The input or mapped genes are compared with genes in co-expression clusters. Similar to expression analysis, if data were input with *P* values, the gene set will be filtered before analysis. Specifically, for inputs with *P* values, gene Manhattan plots are generated by using co-expression clusters as x-axis. Co-expression gene cluster enrichment analysis is performed on the input gene set by using Fisher’s exact test with user selected correction method and cutoff, and the enrichment heatmap is generated accordingly. The enrichment results may differ when using co-expression clusters generated from different expression datasets, and so a Venn diagram is calculated to present the numbers of significantly enriched genes by using different reference data. The average expression quantifications of genes in enriched clusters are calculated and presented as expression heatmaps. The connectivity of input/mapped genes in enriched clusters are counted, and the top 20 genes are treated as core genes. Core genes in the top 5 enriched clusters of each dataset are collected into one network file to construct editable co-expression network graphics.

### Implementation

BEST was developed using a server-client design. The server side was implemented using gunicorn (https://gunicorn.org/), the back-end computing is performed by using Python (version v3.6) and images are generated by using R (version v3.3.2). The client side was built based on the React framework (https://reactjs.org/) and JavaScript libraries Bootstrap (https://getbootstrap.com/) and jQuery (version v3.3.1) (https://jquery.com/). The Python framework flask (http://flask.pocoo.org/) was used to launch the server. All reference data and job information are saved in a MySQL database. The graphical network output is enabled with the plugin Cytoscape (version v3.3.2) [30]. Downloadable results of heatmaps, Venn diagrams and plot diagrams are provided in PNG format. BEST has been tested in most major web browsers such as Chrome, Firefox, and Safari.

## Results

BEST provides a user-friendly user interface. After input data upload and parameter selection, a job can be simply submitted. A job is often finished in several minutes, and the results page will be accessed automatically; however, a user can also retrieve the result later by visiting the results URL link that is generated after job submission. A case example is shown in the “A demo run” module to demonstrate usage.

### Input and parameters

BEST accepts the human gene list and SNP list (with/without *P* value) as input. Genes should be entered with their official symbol. SNP could be entered with their rs ID or in VCF-like and plink-like format. The detail information of input format is shown in “Documents > Tutorial” page. Three types of mapping rules, LD-correlation information of five populations and four types of correction methods could be selected in the SNP to gene mapping. Eight human brain expression data resources could be selected as reference data. Four types of correction methods could be selected the co-expression cluster enrichment too..

### Output

**Fig. 2:**
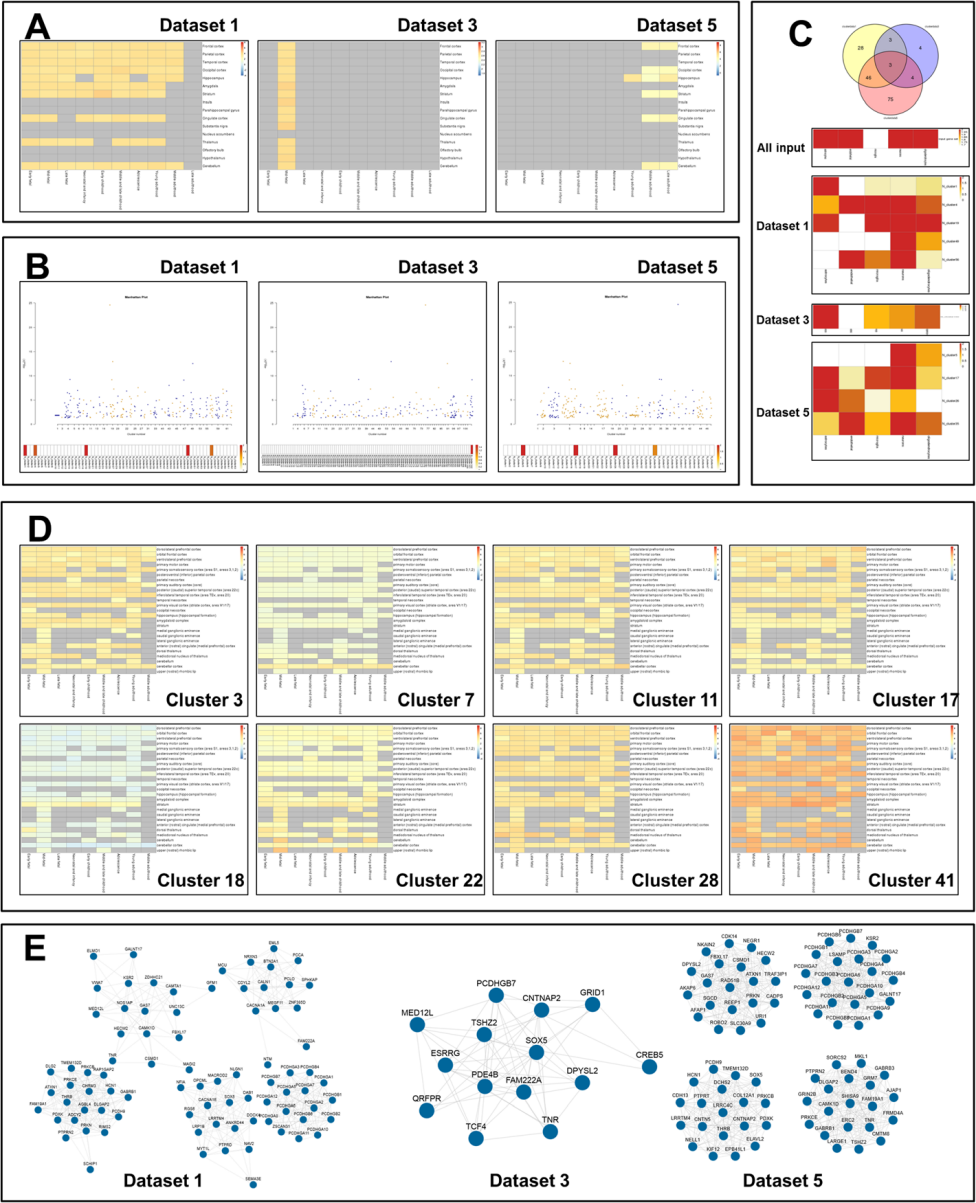
Selected results of the demo run. **a**: Expression heatmap of all inputs; **b**: Cluster-based Manhattan plots and cluster enrichment heatmap; **c**: Enriched gene Venn diagram and cell-type enrichment heatmap; **d**: Expression heatmap of enriched clusters; **e**: Core co-expression network of enriched genes

As shown in Fig. 2, BEST provides heatmaps, Manhattan plots, Venn diagrams, and networks to illustrate the brain spatio-temporal expression patterns of input.

#### Expression heatmap of all inputs

As shown in Fig. 2a, the average expression quantifications of input in all spatio-temporal categories are presented in heatmap form. Although the spatial-temporal categories of each reference dataset are different, BEST generates heatmaps of the same structure (a matrix of 16 brain regions by 10 age stages) to facilitate the comparison between results with reference to different datasets. Specifically, for analysis with reference to RNA-Seq data from Brainspan (dataset 1) and microarray data from the Allen Brain Atlas (dataset 2), heatmaps in the form of matrices of 25 brain regions by 9 age stages and 52 brain regions by 2 age stages are additionally provided, respectively.

#### Cluster-based Manhattan plots and cluster enrichment heatmap

As shown in Fig. 2b, the distribution of inputs in co-expression clusters is presented with a gene-based Manhattan plot, which uses co-expression clusters as x-axis and the negative logarithm of gene-based *P* values as y-axis. The enrichment status of inputs in co-expression clusters is presented as a one row enrichment heatmap.

#### Enriched gene Venn diagram and cell-type enrichment heatmap

The numbers of significantly enriched genes obtained by using different reference datasets are compared in a Venn diagram, as shown in Fig. 2c. It should be noted that the Venn diagram only presents the statistics of results in reference to spatial-temporal categories in a matrix of the 16 brain regions by 10 age stages. The cell-type specific analysis results of all enriched clusters are shown in enrichment heatmaps, with one cluster for each row.

#### Expression heatmap of enriched clusters

Except for average expression quantification of all inputs, BEST calculates the average expression quantifications of genes in each enriched cluster and presents them in expression heatmap form. As shown in Fig. 2d, different gene clusters enriched by the same input gene set may present different spatial-temporal expression patterns.

#### Core co-expression network of enriched genes

The enriched clusters in each dataset are prioritized by corrected *P* values. As shown in Fig. 2e, the core co-expression network of a maximum of the top 5 enriched clusters (if they exist) for each dataset is present as an editable graphic. The core network is constructed by at most 10 input genes (if they exist) with the highest connectivity in each cluster. Moreover, the network files containing co-expression relationships of all input genes in all enriched clusters could be found in the downloaded results folder.

### Usage example

**Fig. 3:**
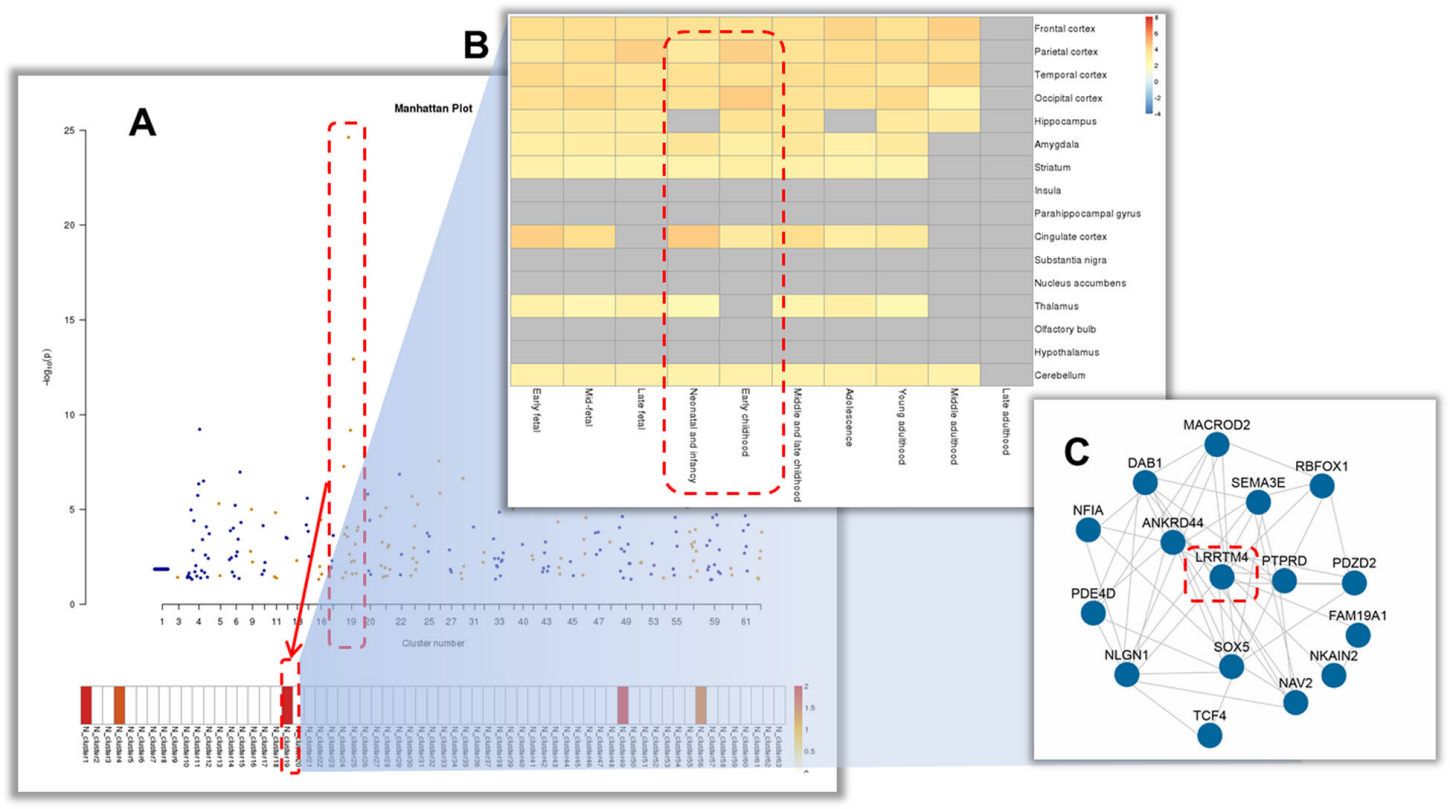
An usage example of BEST. **a**: Cluster-based Manhattan gene plots; **b**: Spatio-temporal expression heatmap; **c**: Gene co-expression network

In the “A demo run” page, we provided a usage example by using the best 10 k SNPs (identified by the original study) of a genome-wide association study (GWAS) of major depressive disorder (MDD) as input [31]. As shown in Fig. 3, the demo data was uploaded with an rs ID (with *P*-value) format. The mapping rule “within gene” is selected for SNP gene mapping; the LD between SNPs is accounted for according to reference data from European populations in gene-based P value calculation. The gene-based P value is further corrected by Bonferroni with the cut off 0.05. Expression dataset 1 (RNA-Seq data from Brainspan), dataset 3 (microarray data from Allen Brain Atlas) and dataset 5 (RNA-Seq data from Xu C et al., 2018) are selected for expression pattern analysis. All of these datasets include co-expression cluster information, and so the Bonferroni correction method and a cutoff of 0.05 are selected for cluster enrichment.

After analysis, 262 genes were mapped from inputs and the detail gene list can be queried from the “Gene_set.txt” in the downloadable results fold. These genes were enriched respectively in five, one and four co-expression clusters from the three selected data resources in the context of 16 by 10 spatio-temporal matrix. Specific for dataset 1, these genes were also enriched in eight clusters in the context of 25 by 9 matrix. These enriched co-expression clusters presented complex spatio-temporal expression features. Take results with reference to dataset 1 in 16 by 10 matrix for example, as shown in Fig. 3a, the most significantly enriched cluster is cluster 19, in which the most significant gene is included. According to the expression heatmap (Fig. 3b), genes in this cluster are expressed in multiple brain regions and a relative up-regulation appears in the stage of neonatal to early childhood (in parietal, occipital, and cingulate cortex). Considering the correlation of early life events and adult depression [32], the epigenetics status of genes in this cluster may be involved in the molecular pathogenesis of MDD. Among the 16 genes enriched in this cluster, the *LRRTM4* gene has the most connections in the co-expression network (Fig. 3c). It may play important role in the cluster-related biological processes.

## Conclusion

BEST is a user-friendly web tool to provide gene expression spatial-temporal pattern analysis in human brain. By using this data tool, users can perform a comprehensive analysis on the results of genetic study (such as GWAS or next-generation sequencing based study); users can also obtain an expression pattern of any genes of interest or SNPs which may have accumulated from previous mechanistic studies or animal models.

In BEST, several human brain expression datasets were compiled and processed as reference data of expression pattern analysis. Gene expression profiling is categorized according to sample brain region and age period. Matrices of expression patterns in the brain regions by age periods will provide more detailed clues on gene function. The cell type specification of gene expression is also considered in BEST, which will further expand the understanding of input-related molecular processes.

Differently from most previous tools, BEST does not focus on the expression status of a single gene: it analyses inputs in a more systematic and comprehensive manner. While BEST treats input as a complete gene set, it also categorizes input into several gene subsets according to the gene expression correlation in the reference dataset. Expression spatial-temporal patterns of such subsets will provide richer and more detailed information, and expression characteristics that are offset by the up- and downregulation in the whole set analysis will be more fully demonstrated. The expression correlations within subsets are presented as a network which will reflect the relationship between analysed genes and intuitively demonstrate the potential importance.

All results in BEST are provided as downloadable graphics, and the data by which the graphics were generated, such as the network text files with a Cytoscape applicable format, are also provided. These result figures are easy to interpret; each graphic illustrates an independent question, and the combination of graphics provides more comprehensive and logical interpretation.

To the best of our knowledge, BEST is the first data tool to support comprehensive human brain spatial-temporal expression pattern analysis; it will facilitate a wide range of human brain related studies. BEST helps to bridge disease-related genetic studies and mechanism studies, provides clues for key gene and molecular system identification, and supports the analysis of disease sensitive brain region and age stages. The analysis results of BEST will further provide evidence for clinical molecular studies related to brain diseases, such as disease biomarker identification and drug development. To provide better supports, BEST will be reviewed annually for possible update. Besides of including new published brain expression profiling as reference data, BEST will attempts to integrate more types of brain functional data, such as including gene expression coupled structural covariance network [33], when relative methods were developed.

## Availability and requirements

**Project name:** BEST

**Project home page:** http://best.psych.ac.cn

**Operating system:** Platform independent

**Programming language:** Python

**Other requierments:** none

**License:** Freely available to academic researchers. Source code available upon request

**Any restrictions to use by non-academics:** BEST use is restricted to academic and non-profit users

## Supplementary information

**Additional file 1: Table S1.** The summary of reference expression data. **Table S2**. The age periods in BEST. **Table S6.** The statistics of co-expression clusters in different datasets

**Additional file 2: Table S3.** The Summary of Spatio-Temporal categories in reference datasets.

**Additional file 3: Table S4.** The addational Spatio-Temporal categories in reference dataset 1.

**Additional file 4: Table S5.** The addational Spatio-Temporal categories in reference dataset 2.

## Data Availability

Source code of BEST is available on GitHub (https://github.com/GuoLiyuan-github/BEST). All data generated or analysed during this study are included in this published article [and its supplementary information files].

## Abbreviations

AFR: African
AMR: American
BH: Benjamini Hochberg
BY: FDR-Benjamini Yekutili
ESA: East Asian
EUR: European
FDR: False discovery rate
GTEx: Genotype-tissue expression
GWAS: Genome-wide association study
HBT: Human Brain Transcriptome
HGNC: Hugo gene nomenclature committee
LD: Linkage disequilibrium
MDD: Major depressive disorder
SAS: South Asian
SNP: Single nucleotide polymorphisms
WGCNA: Weighted correlation network analysis
