# Supplementary material for: BEST: a web server for brain expression Spatio-temporal pattern analysis
Source: BMC Bioinformatics. 2019 Dec 5;20:632. doi: 10.1186/s12859-019-3222-6 (PMC6896511; doi:10.1186/s12859-019-3222-6)
Supplement: Supplementary file 1 — Additional file 1: Table S1. The summary of reference expression data. Table S2. The age periods in BEST. Table S6. The statistics of co-expression clusters in different datasets [file 12859_2019_3222_MOESM1_ESM.docx]

**Supplementary data**

**Table S1.** The summary of reference expression data

**Table S2**. The age periods in BEST

**Table S3.** The Summary of Spatio-Temporal categories in reference datasets.

**Table S4.** The addational Spatio-Temporal categories in reference dataset 1.

**Table S5.** The addational Spatio-Temporal categories in reference dataset 2.

**Table S6.** The statistics of co-expression clusters in different datasets

**Table S1.** The summary of reference expression data

| **Dataset** | **Citation** | **Accession** | **Web interface** | **platform** | **Datatype** |
| --- | --- | --- | --- | --- | --- |
| 1 | Kang et al., 2011 | GSE25219 | <http://www.brainspan.org/rnaseq/search/index.html> | Affymetrix | RPKM |
|  | Johnson et al., 2009 | GSE13344 |  | Affymetrix | RPKM |
|  | Colantuoni et al., 2011 | GSE30272 |  | Illumina Human 49K Oligo array (HEEBO-7 set) | RPKM |
| 2 | Hawrylycz et al., 2012 | Downloaded from web interface | [http://human.brain-map.org](http://human.brain-map.org/) | / | normalized microarray expression values |
| 3 | Miller et al., 2014 | Downloaded from web interface | <http://www.brainspan.org/lcm/search/index.html> | 64K Agilent microarrays | normalized microarray expression values |
| 4 | He Z et al., 2014 | [GSE51264/SRP030628](http://www.ncbi.nlm.nih.gov/geo/query/acc.cgi?acc=GSE51264) | / | Illumina HiSeq 2000 | FPKM/TPM |
| 5 | Xu, C., 2018 | GSE100796/SRP111096 | / | Illumina HiSeq 2000 | FPKM/TPM |
| 6 | He et al., 2017 | [SRP065273](http://www.ncbi.nlm.nih.gov/sra?term=SRP065273) | / | Illumina HiSeq 2000 | FPKM/TPM |
| 7 | Consortium, 2015 | Downloaded from web interface | [https://www.GTExportal.org](https://www.gtexportal.org/) | / | TPM |
| 8 | Lister R et al.,2013,  Schultz MD et al. ,2015 | GSE47966/SRP026048 | / | Illumina HiSeq 2000 (Homo sapiens) | FPKM/TPM |

**Table S2**. The age periods in BEST

| **Period** | **Age** |
| --- | --- |
| Early fetal | 8 PCW<=age<13 PCW |
| Mid-fetal | 13 PCW <= age <24 PCW |
| Late fetal | 24 PCW <= age <38 PCW |
| Neonatal and infancy | 0 M <=age <12 M |
| Early childhood | 1 Y <=age <6 Y |
| Middle and late childhood | 6 Y <= age <12 Y |
| Adolescence | 12 Y <= age < 20 Y |
| Young adulthood | 20 Y <= age <40 Y |
| Middle adulthood | 40 Y <=age <60 Y |
| Late adulthood | 60 Y <= age |

PCW: postconceptional week

M: month

Y: year

**Table S6.** The statistics of co-expression clusters in different datasets

| **Dataset** | **Cluster number** | |
| --- | --- | --- |
| 1 | *In matrix of 16*10* | 63 |
|  | *In matrix of 25*9* | 45 |
| 2 | *In matrix of 16*10* | 32 |
|  | *In matrix of 51*2* | 35 |
| 3 |  | 102 |
| 5 |  | 48 |
| 7 |  | 11 |
